# Clinical and imaging characteristics of growing skull fractures in children

**DOI:** 10.1038/s41598-024-56445-z

**Published:** 2024-03-07

**Authors:** Qingshuang Zhao, Jianbin Ying, Yehuang Chen, Fan Chen, Taotao Zhang, Junjie Jing

**Affiliations:** 1 Department of Neurosurgery, Fujian Children’s Hospital (Fujian Branch of Shanghai Children’s Medical Center), College of Clinical Medicine for Obstetrics and Gynecology and Pediatrics, Fujian Medical University, Fuzhou, Fujian People’s Republic of China; 2 Department of Neurosurgery, The 900th Hospital of the Joint Logistic Support Force, Fuzhou, Fujian People’s Republic of China

**Keywords:** Growing skull fracture, Classification, Children, Treatment, Paediatric research, Brain injuries

## Abstract

Growing skull fracture (GSF) is an uncommon form of head trauma among young children. In prior research, the majority of GSFs were typically classified based on pathophysiological mechanisms or the duration following injury. However, considering the varying severity of initial trauma and the disparities in the time elapsed between injury and hospital admission among patients, our objective was to devise a clinically useful classification system for GSFs among children, grounded in both clinical presentations and imaging findings, in order to guide clinical diagnosis and treatment decisions. The clinical and imaging data of 23 patients less than 12 years who underwent GSF were retrospectively collected and classified into four types. The clinical and imaging characteristics of the different types were reviewed in detail and statistically analyzed. In all 23 patients, 5 in type I, 7 in type II, 8 in type III, and 3 in type IV. 21/23 (91.3%) were younger than 3 years. Age ≤ 3 years and subscalp fluctuating mass were common in type I–III (P = 0.026, P = 0.005). Fracture width ≥ 4 mm was more common in type II–IV (P = 0.003), while neurological dysfunction mostly occurred in type III and IV (P < 0.001).Skull “crater-like” changes were existed in all type IV. 10/12 (83.3%) patients with neurological dysfunction had improved in motor or linguistic function. There was not improved in patients with type IV. GCS in different stage has its unique clinical and imaging characteristics. This classification could help early diagnosis and treatment for GCS, also could improve the prognosis significantly.

## Introduction

Growing skull fracture (GSF) is a rare type of head injury in children, mainly occurring in children under 3 years old, with an incidence rate of 0.05–1.6%^1,2^. GSF is mostly caused by closed cerebral injury, often accompanied by scalp hematoma. Linear skull fracture and dural tear are the pathological basis^3,4^. Current studies believe that a fracture line width greater than 4 mm, local subdural effusion, and age less than 3 years are predictors of early diagnosis^5^. GSF can cause encephalocele, hydrocephalus encephalomalacia, and other secondary damage, resulting in serious neurological dysfunction^6^.

Ito et al.^7^ divided GSF into three types according to different herniations: cystic type, granulomatous type and mixed type. According to the pathophysiological process, Ziyal et al.^8^ divided GSF into four stages: fracture formation and dural tear in the first stage, arachnoid or pia herniation in the second stage, encephalomalacia cyst formation in the third stage, and ventricular perforation deformity in the fourth stage. These classifications mainly based on pathological and pathophysiological changes after injury. Liu et al.^9^ divided GSF into three stages based on clinical manifestation and time from injury: the first stage was from injury to before fracture line expansion, and the postoperative prognosis of this stage was good; the second stage started from fracture line enlargement until 2 months after injury, and some children had residual neurological dysfunction in this stage. The third stage was defined as more than 2 months after injury and was usually accompanied by severe neurological dysfunction. A lot of reports on GSF followed these classifications, but the patients were usually in latest stage^10–12^.

Due to the different severity of the initial injury, the progress of the disease is different, and it is obviously limited to classified by the time from injury. Different time from injury to hospital is also very different, so it is more clinical to classify them according to clinical and imaging features. The aim of this study was to present a useful classification for GSF based on clinical and imaging characteristics to refine previous classification, and improve diagnosis and prognosis.

## Materials and methods

The clinical data of patients who were diagnosed GSF in Fujian Children’s Hospital and the 900th Hospital from March 2013 to January 2022 were retrospectively collected. The inclusion criteria: (1) age less than 12 years; (2) underlied head trauma; (3) skull fractures and dural tear were found in operation; and (4) the availability of complete clinical data. In this study, we conducted a retrospective review of the patients' data, extracting and analyzing detailed information on age, gender, symptoms and signs, radiological findings, surgical interventions, post-surgical complications, and overall prognosis.

All cases were categorized into four types based on clinical and imaging characteristics. Type I represents the early stage, characterized by a linear skull fracture with torn dura following closed head injury. Type II corresponds to the developmental stage, defined by progressive changes including bone deficit, enlarged scalp mass, and subdural effusion. Type III represents the deteriorating stage, characterized by continuous brain tissue injury resulting from a growing skull fracture. Finally, Type IV represents the stable stage, exhibiting bone deficit and necrotic cyst in the brain.

The clinical and imaging features were recorded as categorical data, and statistical analysis was conducted using SPSS 27. Ridit analysis was utilized to assess and compare the differences across the four types.

The study was performed with the ethical standards of the Helsinki declaration. The study protocol was approved by Fujian Children’s Hospital ethics committee(2022ETKLR12041).

### Ethical approval and consent to participate

The study was performed with the ethical standards of the Helsinki declaration. Due to the retrospective nature of our study, the Fujian Children’s Hospital ethics committee waived the need for informed consent of the participating patients(2022ETKLR12041).

## Results

### Clinical and imaging characteristics

A total of 23 patients met the criteria, with 15 being male and 8 being female. The mean age of these patients was 1.7 years, ranging from 1 month to 11 years. Notably, 21 patients (91.3%) were younger than 3 years old. All patients underwent CT three-dimensional reconstruction before the operation, and 21 patients underwent MRI examination. All patients had skull fractures; there were 20 cases of linear fractures and 3 cases of local skull defects. The time from injury to operation was 1 day to 6 years. The causes of injury included falling in 18 cases and traffic accidents in 5 cases.

Subscalp fluctuating mass is common in all type I, II and III. 8 (34.8%) were associated with epileptic seizures, 2 in type II and 6 in type III. Neurological dysfunction were existed in 13 (56.5%) patients, 2 in type II showed contralateral limb weakness, 8 in type III showed hemiplegia and aphasia, 3 in type IV presented with contralateral limb weakness or hypermyotonia.

From CT and MRI, 19 (82.6%) showed fracture width ≥ 4 mm, include 1 in type I, and all in type II, III and IV. 10 (43.5%) showed hernia of brain tissue, 2 in type II and all type III. 7 (30.4%) suffered hydrocephalus, 2 in type II and 5 in type III. Subdural effusion (16/23, 69.5%) were observed in 1 of type I and all type II and III. Skull "crater-like” changes were existed in all type IV (Table 1).

**Table 1:** Clinical and imaging characteristics of each type.

	N (%)	Type I	Type II	Type III	Type IV	Hc	P
Total	23 (100.0)	5 (21.7)	7 (30.4)	8 (34.8)	3 (13.1)		
Sex							
Male	15 (65.2)	3	5	6	1	0.330	0.742
Female	8 (34.8)	2	2	2	2		
Age							
≤ 3 years	21 (91.3)	5	7	8	1	2.229	0.026
> 3 years	2 (8.7)	0	0	0	2		
Subscalp fluctuating mass							
Yes	20 (86.9)	5	7	8	0	2.798	0.005
No	3 (13.1)	0	0	0	3		
Epilepsy							
Yes	8 (34.8)	0	2	6	0	1.385	0.166
No	15 (65.2)	5	5	2	3		
Neurological dysfunction							
Yes	13 (56.5)	0	2	8	3	3.801	< 0.001
No	10 (43.5)	5	5	0	0		
CT and MRI							
Fracture width ≥ 4 mm							
Yes	19 (82.6)	1	7	8	3	2.983	0.003
No	4 (17.4)	4	0	0	0		
Hernia of brain tissue							
Yes	10 (43.5)	0	2	8	0	1.901	0.057
No	13 (56.5)	5	5	0	3		
Hydrocephalus							
Yes	7 (30.4)	0	2	5	0	1.126	0.260
No	16 (69.6)	5	5	3	3		
Subdural effusion							
Yes	16 (69.6)	1	7	8	0	0.410	0.682
No	7 (30.4)	4	0	0	3		

### Classification based on clinical and imaging characteristics

**Figure 1:**
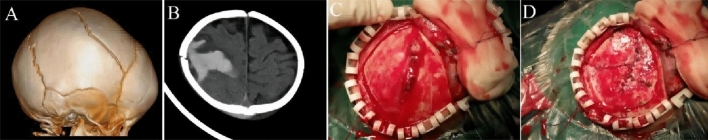
The imaging characteristics and intraoperative findings of Type I. (**A**) In 3D-CT, a long linear fracture, no obvious separation of the fracture line. (**B**) Cerebral contusion and cerebral hematoma below the fracture. (**C**) The dural membrane was damaged, and brain tissue contusion was highlighted on the dural surface of the brain. (**D**) After the hematoma was removed, the dura mater was tightly sutured, and the bone flap was reset and fixed.

Type I (early stage): sub scalp hematoma and progressive enlargement were the common. CT scan showed mainly presented with long linear skull fractures (often less than 4 mm in width), accompanied by cerebral contusion and laceration or intracerebral hematoma, and the location of contusion and laceration was consistent with the fracture line, and MRI showed linear fracture of the skull, brain contusion or brain hematoma, fluid signals under the scalp (Fig. 1).

**Figure 2:**
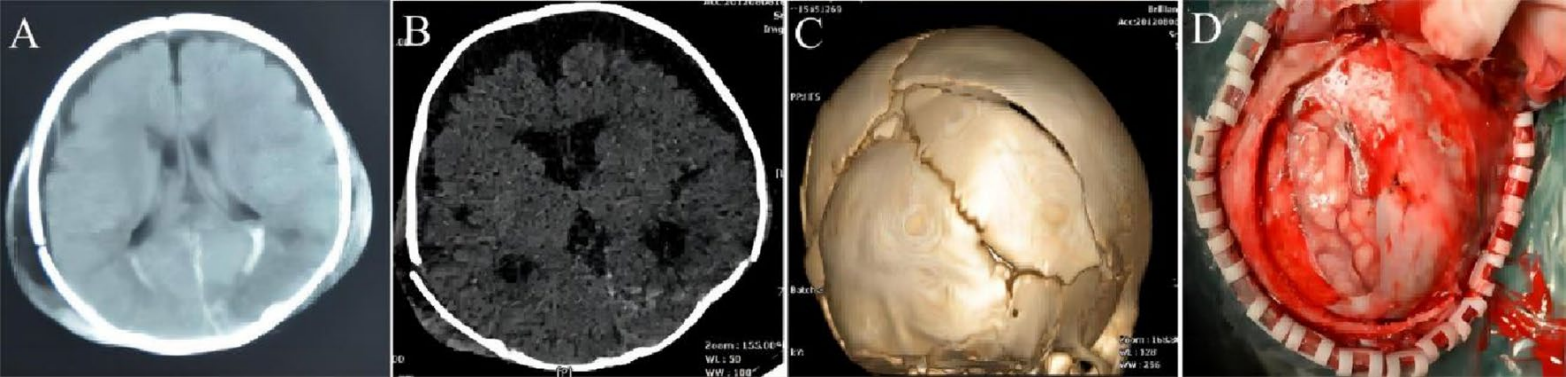
The imaging characteristics and intraoperative findings of Type II. (**A**) One day after the injury, CT indicated a linear fracture of the right parietal bone and subscalp hematoma and a large number of low-density shadows under the skin. (**B**) Nine days after the injury, CT showed local subscalp effusion, and bilateral subdural effusion. (**C**) 3D-CT showed a linear parietal bone fracture, fracture width greater than 4 mm. (**D**) The dural defect was obvious and not below the fracture line but below the bone flap, the brain tissue and the dural rim; after the adhesions were released, the subdural effusion was released.

Type II (developmental stage): fluctuating mass under the scalp enlarged progressively, and few patients took focal neurological dysfunction. CT showed a long linear skull fracture or comminuted depression fracture, the fracture line width could be greater than 4 mm, a large number of low -density shadows could be seen subcutaneously, and subdural effusion near the fracture line could be seen intracranially. MRI showed subcutaneous fluid signals, brain tissue adhesion to the fracture line or slight herniation of the fracture line, subdural fluid accumulation in the source of brain tissue adhesion or hernia, and mild hydrocephalus (Fig. 2).

**Figure 3:**
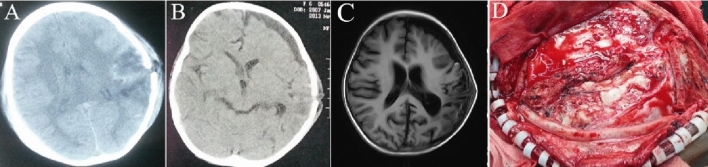
The imaging characteristics and intraoperative findings of Type III. (**A**) On the first day after injury, CT showed a linear fracture of the left temporoparietal bone and a cerebral contusion below the fracture line. (**B**) On the 19th day after the injury, CT showed a linear fracture of the left temporoparietal bone, the cerebral contusion below the fracture line was better than before, and local subdural effusion had formed. (**C**) On the 41st day after the injury, the MRI showed a fracture line herniation of the left temporoparietal brain tissue, local cystic changes and necrosis of the brain tissue, and local subdural effusion. (**D**) Extensive dural defects, contusion, local brain tissue contusion and necrosis were observed during the operation.

Type III (developmental stage): patients had severe neurological dysfunction, such as hemiplegia, aphasia, epilepsy, etc. CT showed a wide fracture line, usually more than 4 mm wide, and MRI images showed obvious brain tissue and/or cerebrospinal fluid herniation at the fracture line, accompanied by severe subdural effusion and even midline structural displacement; sometimes severe hydrocephalus was in evidence as well (Fig. 3).

**Figure 4:**
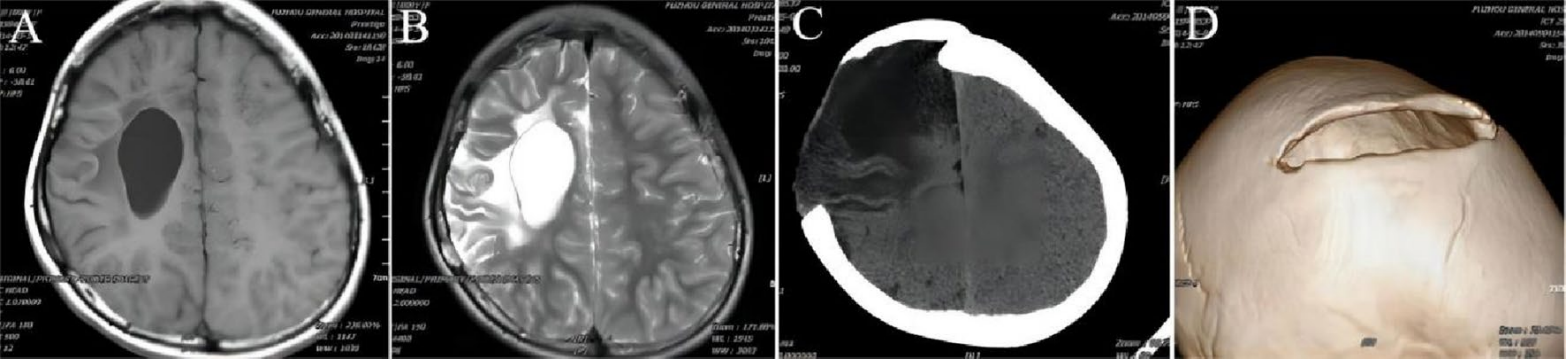
The imaging characteristics of Type IV. (**A**) MRI-T1WI showed lateral ventricle expansion. (**B**) MRI-T2WI showed atrophy and necrosis of the right parietal brain tissue. (**C**) CT showed a softening of the right parietal brain tissue, a local skull defect. (**D**) 3D-CT showed a “crater-like” change at the defect margin.

Type IV (stable stage): patients suffered permanent neurological dysfunction, such as paraplegia, aphasia, increased muscle tension, epilepsy, etc. CT and MRI showed local skull defects, thickened and everted skull edges, “crater-like” changes, encephalomalacia or cystic changes in brain tissue, and ventricular perforation malformation in corresponding parts (Fig. 4).

### Surgical treatment

In patients with type I–III GSF, bone flaps were removed from both sides along the fracture line. The longer the time after injury, the larger the range of bone flaps that were seen. The extent of dural defects were completely exposed, adhesions between the dura and brain tissue were released, subdural effusion was released, and the subdural space was rinsed to remove part of the dead brain tissue. Then the dura was tightly sutured. In the cases of large defects, the bone flap was tightly repaired with its own periosteum or artificial dura, and the bone flap was fixed and restored with silk thread or absorbable skull lock. Because the dura of children with type IV GSF had been cicatricized and was closed by itself, only skull repair was needed. During surgery, the new “crater” bone was removed, and the procedure was followed by plastic repair with a titanium plate. Seven patients (2 in type II, 5 in type III) accepted VP shunt.

### Clinical prognosis

All the patients were followed up for 0.5 to 3 years, and there was no death or aggravation of neurological dysfunction. 12 Patients (4 in type I, 8 in type II) with neurological dysfunction were treated by rehabilitation and hyperbaric oxygen for 3 months after operation. 10 (4 in type I, 6 in type II) had improved in motor or linguistic function. There was not improved in patients with type IV.

## Discussion

In 1816, John Howship^13^ first reported skull fracture growth in children and realized that the mechanism was linear skull fracture accompanied by dural lacerations and brain tissue or arachnoid herniation from the defective dura and fracture line, leading to gradual expansion of the fracture line. Studies have shown that the development of GSF is also affected by a variety of factors, such as the following: (1) After the brain tissue adheres to the dura mater, the local cerebrospinal fluid circulation is impaired, causing subdural effusion or hydrocephalus, increasing intracranial pressure, further pushing and herniating the brain tissue, and hindering fracture healing^14^. (2) Ischemia and scar formation at the broken end of the fracture, which can no longer heal itself^15^. (3) Since osteogenesis in children depends on the periosteum and dura, when the space between the skull and periosteum is occupied by herniated cerebrospinal fluid or brain tissue, the dura on the inner surface of the skull is also defective, resulting in skull osteogenesis failure^16^. These developments can lead to the further expansion of the fracture line and introduce problems to be solved in surgical treatment.

The pathological basis of GSF is fracture accompanied by dural tear. Early diagnosis is still a difficult problem. Head 3D-CT scan can confirm the length and width of the fracture line, and the diagnosis can be completed with MRI when brain tissue is adhered to or herniated with the damaged dura mater or when there is subdural effusion, but these images are usually seen more than 1 week after trauma. Matsuura et al.^17^ reported enhanced MRI was used to display the edge of the dural defect on the 10th day after injury. However, whether enhanced MRI can identify dural damage at the early stage after injury has not been reported.

Early diagnosis and treatment of GSF is key to achieving a good prognosis. Fracture line width greater than 4 mm, local subdural effusion, and age less than 3 years of age are considered predictors of early diagnosis^18^. However, due to the lack of early understanding of the disease, some reports in the literature are latest cases. Age less than 3 years is an important indicator of GSF after trauma, which has been agreed upon, but the fracture width ≥ 4 mm, not necessarily in the early stage. In this study, fracture width ≥ 4 mm was more common in type II–IV (P = 0.003). 3 patients in type I showed fracture width less than 4 mm. Singh et al.^19^ found that MRI indicated that dural defects accompanied by intracerebral hematoma were a high-risk factor for growth fracture, and no GSF was found in patients without dural damage or intra- cerebral hematoma after follow up.

Based on pathological changes, Ito et al.^7^ introduced the initial classification of growing skull fractures (GSF) in 1977, categorizing them into cystic, granulomatous, and mixed types. Rahman revised the classification in 1994, dividing GSF into three types based on brain pathology. However, Rahman's classification can be confusing due to brain lesion evolution. SinghI et al.^19^ reported that Rahman's classification often led to mixed types in clinical practice. With the use of CT and MRI, the dynamic evolution of GSF has become apparent. Liu et al.^9^ introduced a three-stage classification system in 2012 that was more pragmatic and based on clinical manifestations and post-injury duration. While this system provided clarity, it assumed a homogeneity in the progression of growing skull fractures (GSFs) that may not accurately reflect the realities of variable patient conditions. Factors such as patient age, severity of injury, and skull fracture location can significantly influence the progression of GSFs. Specifically, the second stage in their system spanned a prolonged period from fracture line enlargement to two months post-injury, which was considered unsuitable due to residual neurological dysfunction reported in some children during this time. Given the varying severities of initial injuries and the diverse progression among patients, grouping them into a singular stage may not be appropriate.

When the fracture line begins to expand, there is still less neurological dysfunction in the early stage, until the brain content continues to herniate and the fracture line is further expanded, the neurological dysfunction will become more and more serious. Our results showed neurological dysfunction were existed in 13 (56.5%) patients, 2 in type II, 8 in type III, 3 in type IV, neurological dysfunction mostly occurred in type III and IV (P < 0.001). These results support us to update the classification of GSF.

Type III GSF is the progressive neurological deterioration stage. In this stage, patients still need to undergo surgery to release adhesion and repair the dura. Neurological function can still be improved to a certain extent after surgery. Type IV GSF is stable, some children with type II or even type I do not show progressive deterioration of neurological function but gradually stabilize with the prolongation of post- injury time, with only progressive expansion of the fracture line and development of large skull defects, dural defects and pseudodural formations. Only skull repair is needed. Artificial materials or autologous bone from corresponding parts can be used for skull repair^20^.

The surgical treatment of growing fractures is basically consistent in various reports. It should be noted that the dural defect is likely not below the fracture line but below the bone flap next to the fracture line. The longer the postinjury time before operation, the greater the extent of the dural defect. Singhal et al.^21^ reported that the extent of dural defects was up to twice that of bone defects. Therefore, it is recommended that the extent of bone flap opening should be large enough to completely expose the dural defect^22^.

In addition to the treatment of complications such as subdural effusion and hydrocephalus, anti-epileptic treatment, hyperbaric oxygen and rehabilitation therapy should be considered. For those with severe neurological dysfunction, hyperbaric oxygen and rehabilitation therapy can help children to be better recovery.

## Conclusions

According to the clinical and imaging characteristics, GSFs were divided into 4 types that can guide treatment and help prognosis assessment. Early diagnosis and treatment of skull fracture growth can significantly improve the prognosis for children.

## Data Availability

Data in this study are available from the corresponding author on reasonable request.
